# A novel deletion mutation of the EXT2 gene in a large Chinese pedigree with hereditary multiple exostosis

**DOI:** 10.1054/bjoc.2001.1880

**Published:** 2001-07

**Authors:** C Y Xiao, J Wang, S Z Zhang, W Van Hul, W Wuyts, W M Qiu, H Wu, G Zhang

**Affiliations:** Department of Medical Genetics, West China Medical Center, Sichuan University, Chengdu, 610041, P. R. China; Key Laboratory of Morbid Genomics and Forensic Medicine, Sichuan Province, Chengdu, 610041, P. R. China; Department of Medical Genetics, University of Antwerp, Antwerp, 2610, Belgium

**Keywords:** hereditary multiple exostoses, EXT2, deletion mutation, chondrosarcoma

## Abstract

Hereditary multiple exostoses (EXT) is an autosomal dominant disease characterized by the formation of cartilage-capped prominences (exostoses) that develop from the juxta-epiphyseal regions of the long bones. 3 genes are known to be involved in the formation of exostoses. Among them, EXT1 and EXT2, which encode enzymes that catalyse the biosynthesis of heparan sulfate, an important component of the extracellular matrix, are responsible for over 70% of the EXT cases. A large Chinese family with hereditary multiple exostoses has been analysed and the disease-causing mutation has been found. Blood samples were obtained from 69 family members, including 23 affected individuals. The EXT phenotype was shown to be linked to the EXT2 gene by using 2-point linkage analysis. After polymerase chain reaction (PCR)-single strand conformation polymorphism (SSCP) analysis and DNA sequencing, a previously unreported deletion of a G in exon 3 of EXT2 gene was observed. This deletion co-segregated with the disease phenotype, suggesting that it is the disease-causing mutation in this family. Furthermore, in at least 4 members chondrosarcoma occurred after either an operation or injury of the exostosis and 3 of them died of the malignance in the family. Whether the operation or injury was responsible for the malignant transformation still needs further study. © 2001 Cancer Research Campaign http://www.bjcancer.com


*British Journal of Cancer* (2001) **85**(2), 176–181
© 2001 Cancer Research Campaign
doi: 10.1054/ bjoc.2001.1880, available online at http://www.idealibrary.com on IDEAL®

http://www.bjcancer.com

### A novel deletion mutation of the EXT2 gene in a large Chinese pedigree with hereditary multiple exostosis

**CY Xiao[1,2], J Wang[1,2], SZ Zhang[1,2], W Van Hul[3], W Wuyts[3], WM Qiu[1,2], H Wu[1,2] and G Zhang[1,2]**

[1]Department of Medical Genetics, West China Medical Center, Sichuan University, Chengdu, 610041, P. R. China; [2]Key Laboratory of Morbid Genomics and Forensic Medicine, Sichuan Province, Chengdu, 610041, P. R. China; [3]Department of Medical Genetics, University of Antwerp, 2610 Antwerp, Belgium

**Summary** Hereditary multiple exostoses (EXT) is an autosomal dominant disease characterized by the formation of cartilage-capped prominences (exostoses) that develop from the juxta-epiphyseal regions of the long bones. 3 genes are known to be involved in the formation of exostoses. Among them, EXT1 and EXT2, which encode enzymes that catalyse the biosynthesis of heparan sulfate, an important component of the extracellular matrix, are responsible for over 70% of the EXT cases. A large Chinese family with hereditary multiple exostoses has been analysed and the disease-causing mutation has been found. Blood samples were obtained from 69 family members, including 23 affected individuals. The EXT phenotype was shown to be linked to the EXT2 gene by using 2-point linkage analysis. After polymerase chain reaction (PCR)-single strand conformation polymorphism (SSCP) analysis and DNA sequencing, a previously unreported deletion of a G in exon 3 of EXT2 gene was observed. This deletion co-segregated with the disease phenotype, suggesting that it is the disease-causing mutation in this family. Furthermore, in at least 4 members chondrosarcoma occurred after either an operation or injury of the exostosis and 3 of them died of the malignance in the family. Whether the operation or injury was responsible for the malignant transformation still needs further study. © 2001 Cancer Research Campaign http://www.bjcancer.com

**Keywords**: hereditary multiple exostoses; EXT2; deletion mutation; chondrosarcoma

Hereditary multiple exostosis (HME, also called EXT) is an autosomal dominant disorder characterized by the presence of multiple exostoses (osteochondromas) localized mainly in the juxta-epiphyseal region of long, tubular bones. These osteochondromas are apparent during the first decade of life in more than 80% of the patients (Solomon, 1963). The estimated prevalence of EXT ranges from 1.3/100 000 (Hennekam, 1991) to 2/100 000 (Schmale et al, 1994). Penetrance is estimated between 66% (Suyiura et al, 1976) and 100% (Wicklund et al, 1995). An exostosis can transform into a chondrosarcoma or an osteosarcoma in about 0.9% to 2.9% of EXT-patients. Other features include short stature, limb length inequalities, nerve and blood vessel compression, impaired articular function, bowing of tubular bones and synostoses (Wicklund et al, 1995).

EXT is a genetically heterogeneous disease with at least 3 susceptibility loci. 2 of them, the EXT1 gene on chromosome 8q24.1 and the EXT2 gene on chromosome 11p11 have been isolated by positional cloning (Ahn et al, 1995; Stickens et al, 1996; Wuyts et al, 1996). Since loss of heterozygosity (LOH) has been observed with markers linked to the EXT1 or EXT2 genes in both sporadic and exostoses-derived tumour materials, it has been suggested that the EXT genes might be tumour suppressor genes (Hecht et al, 1995; Raskind et al, 1995). The EXT3 gene has been mapped to 19p (Le Merrer et al, 1996). In addition, 3 EXT-like genes have been mapped, namely EXTL1 on 1p36 (Wise et al, 1997), EXTL2 on 1p11–12 (Wuyts et al, 1997) and EXTL3 on 8p12–22 (Van Hul et al, 1998). They all encode products with the homologue C-terminals. Although the EXTL genes are considered strong candidate genes for EXT, most of the EXT patients are linked with EXT1 and EXT2 and so far no EXTL mutation has been detected in any EXT patient.

We now analysed a large Chinese EXT pedigree with at least 38 affected members. After linkage analysis and mutation analysis, a novel mutation, namely, a deletion of one nucleotide in exon 3 of the EXT2 gene was observed.

### MATERIALS AND METHODS

### EXT family

Blood or saliva specimen were collected from 69 members of a Chinese multigeneration pedigree with hereditary multiple exostoses with the informed consent of the participants. Among them, 23 were affected.

Genomic DNA was extracted from peripheral blood lymphocytes by salting-out method (Miller et al, 1988), or from mouth swabs by Chelex method (Walsh et al, 1991). Mouth swabs were collected by firm rubbing of a cotton bud backwards and forwards between the cheek and gum several times (3 cotton buds were used for each individual).

### Linkage analysis

Linkage analysis was carried out using the LINKAGE 5.1 package program MLINK (Lathrop et al, 1984) for 2-point analysis, assuming dominant inheritance with full penetrance and EXT frequency set to 1/50 000. Microsatellite repeat markers were

*Received 20 September 2000*
*Revised 22 February 2001*
*Accepted 29 March 2001*

*Correspondence to:* SZ Zhang selected according to literature (Tang et al, 1998). D8S85, D8S285 and D8S199 from 8q24.1 (EXT1), D11S903, D11S1361 and D11S1355 from 11p11 (EXT2) and D19S221 from 19p(EXT3) were studied.

### Mutational screening

After linkage was suggested to the EXT2 gene, exons 2 to 14 of the EXT2 gene were amplified by using PCR profiles from Wuyts et al (1998) with slight modification.

The sequences of the primers were shown in Table 1. Exon 2 was split into 3 overlapping fragments to obtain amplification products suitable for SSCP analysis. PCR was performed in a 25 μl reaction volume, containing 50–100 ng of genomic DNA, 1 × PCR buffer, 200 μM of each dNTP, 0.4 μM of each primer pair, 1.5 mM of $MgCl_2$ and 1 unit of Taq DNA polymerase (GENETAQ). PCR was carried out in Perkin Elmer 9600 thermal cycler. After predenaturation at 95°C for 5 min, the PCR was carried out for 30–35 cycles of denaturation at 94°C for 30 s, annealing at suitable temperature for 30 s, extension at 72°C for 30 s, and a final extension at 72°C for 5 min. The annealing temperature was 55°C for all primer pairs, with the exception of primer pairs amplifying exons 3, 5 and 10, for which the annealing temperature was changed to 51°C. The amplified products were then subjected to agarose gel eletrophoresis to judge their amplification efficiency. Finally, SSCP of the amplified products was performed according to standard protocol, and the gel was silver stained and analysed.

The PCR fragments containing aberrant fragments (i.e. exon 3) were reamplified, and then either purified by use of PCR Clean Up Kit (Boehringer Mannheim) and directly sequenced by using Cy5 dye-terminator thermal sequenase sequencing kit (Pharmacia Biotech); or cloned into pGEM T-easy vector (Promega), and the identified double strand plasmids were then sequenced by using ABI Prism DNA sequencing kit (PE). Sequencing reactions were performed according to the instructions by the kit supplier. For PCR products sequencing, one of the amplification primers for exon 3 was used as sequencing primer, and sequences were analysed on ALF express automatic DNA sequencer (Pharmacia Biotech); for plasmid sequencing, M13 reverse primer was used as sequencing primer, and sequences were analysed on AB1 377 automatic DNA sequencer (PE).

### RESULTS

### Clinical description of the family

In the whole family, according to the anamnesis of some family members, at least 35 individuals were severely affected with multiple large exostoses, some of them were more than 10 cm in diameter, 3 (V51, V56 and VI25 in Figure 1) were slightly affected, with only a isolated small exostosis less than 1 cm in diameter. Among the severely affected at least in 4 individuals including the proband (IV53, the first patient observed by doctor in the family) an exostosis has transformed into chondrosarcoma. The pedigree is shown in Figure 1. The diagnosis of the disease in the family was made by osteologists at the First Affiliated Hospital of West China University of Medical Sciences. Some of the patients have been confirmed by regional X-radiographic analysis. In addition, the proband's diagnosis was made also by biopsy of

**Table 1** Primers and related parameters used in the study

| Name | Amplified exon | Sequence | Product length (bp) | Annealing temperature |
|---|---|---|---|---|
| EXT2-ex2a | 2 | ctctcccctggtgacc | 338 | 55°C |
| EXT2-ex2A8 | | *CACAGCGATAGACATCAAAACACG* | | |
| EXT2-ex2A26 | 2 | GACAGTCCCATCCCAGAGCGG | 249 | 55°C |
| EXT2-ex2A25 | | *GGAGGGAACAAACAGACAGG* | | |
| EXT2-ex2A4 | 2 | ACTACACTGATGACATCAACCG | 176 | 55°C |
| EXT2-ex2b | | *ccctttagttccctgagggcc* | | |
| EXT2-ex3a | 3 | gttgacacattaattctccc | 184 | 51°C |
| EXT2-ex3b | | *gaacaaaaatgatcttgaaccc* | | |
| EXT2-ex4a | 4 | gaataaagtcctttctttctcatcg | 205 | 55°C |
| EXT2-ex4b | | *cagtaaaggcacacctggc* | | |
| EXT2-ex5a | 5 | gcaattttccaatcacctg | 267 | 51°C |
| EXT2-ex5b | | *cctgagcctttgcgagagg* | | |
| EXT2-ex6a | 6 | ctagtttgtaatctcttgcctc | 222 | 55°C |
| EXT2-ex6b | | *tacgcagaaccactaatgtagag* | | |
| EXT2-ex7a | 7 | gggatgtggggctgaaggagg | 293 | 55°C |
| EXT2-ex7b | | *ctcctgtccctctgtatccagtc* | | |
| EXT2-ex8a | 8 | gcttgctcacttaaaacagc | 195 | 55°C |
| EXT2-ex8b | | *gcctcatgtggctagcac* | | |
| EXT2-ex9a | 9 | cagctgcttttctgacccg | 263 | 55°C |
| EXT2-ex9b | | *gatccagctgagagaggcac* | | |
| EXT2-ex10a | 10 | cctcacaaaagttaggag | 240 | 51°C |
| EXT2-ex10b | | *aaacacacctgtgtaaaacc* | | |
| EXT2-ex11a | 11 | gaatggttgctgtctgaattggg | 235 | 55°C |
| EXT2-ex11b | | *ctcagttttgtcaccttgcc* | | |
| EXT2-ex12a | 12 | ccccttatttatcagctaaaggg | 220 | 55°C |
| EXT2-ex12b | | *caagtgagtggcagagcc* | | |
| EXT2-ex13a | 13 | gtccttgacactgacagccagg | 175 | 55°C |
| EXT2-ex13b | | *tagagatcagaggctaaggcgc* | | |
| EXT2-ex14a | 14 | caaacccctcctccccacctcctc | 318 | 55°C |
| EXT2-ex14c | | *GTGGGTTAGGTGGGTGCATGCC* | | |

Reverse primers are italicized. Primers located in introns are lower-cased, primers located in exons are capitalized.

© 2001 Cancer Research Campaign

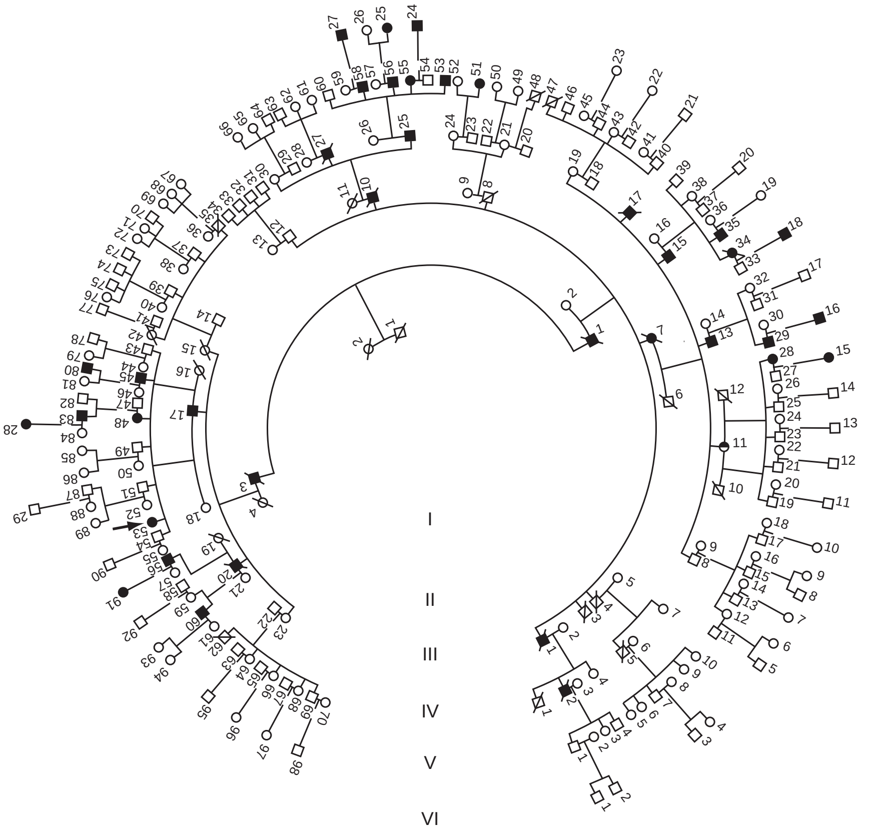

**Figure 1** Pedigree of the present study. Arrow indicates the proband (IV53) (the first patient observed by doctors). All the open boxes represent healthy males, and open circles healthy females. The half-filled circle (IV11) represents a carrier, i.e., who carries the mutant gene but the phenotype is normal. Boxes or circles with a crossing line mean that the person has already died the exostosis. Clinically all the severely affected members were of short stature with mean height of less than 155 cm in male patients, and had multiple exostoses at the metaphyses of the long tubular bones, some of them even had 30–40 exostoses all over the body. The local appearance and radiological findings in some severely affected members are shown in Figure 2. The proband noticed her first exostosis of about 1 cm in diameter at her left pubis when she was 33 years old. The exostosis grew slowly to about 2 cm 2 years later. Then an operation was performed to remove this exostosis and biopsy of the removed exostosis was performed. But 1 year after, the exostosis recurred, grew rapidly and became painful, then the tumour malignantly transformed to well-differentiated chondrosarcoma, which was confirmed by biopsies of the removed tissues at least 7 times during the following years. The chondrosarcomas then metastasized to the soft tissues close to spinal column of abdomen and several other sites in her pelvis, so an extended hemipelvectomy was performed. In the following 11 years she has had more than 7 operations to remove the recurred chondrosarcomas. Similarly malignant transformation in another 3 severely affected members had occurred after accidental injury of one of the exostosis.

### Linkage analysis

All the 3 markers from 11p11 (EXT2) gave a 2-point LOD-score value of >3 and showed no recombination between the markers and the EXT phenotype, therefore the linkage between the exostoses and EXT2 gene was firmly established in the large family. The markers from 8q24 and 19p gave very low LOD-score of <1, so the linkage to EXT1 and EXT3 can be ignored. According to this result, the family's EXT is linked to EXT2 was concluded.

### Mutation analysis

SSCP analysis of exon 3 of all the family members revealed that all severely affected members showed the same pattern with 2 additional single-strand bands as compared with the unaffected members and the unrelated healthy members in the family, suggesting that in the severely affected patients the 2 alleles of the exon 3 of EXT2 gene are different. However, in 2 of the slightly affected individuals (V 51 and VI 25), no additional single strand bands were observed. Figure 3 shows SSCP results of some of the members in the family.

Exon 3 of 3 different severely affected members, one slightly affected member and one unrelated healthy individual were sequenced. In all the 3 severely affected patients, there was a deletion of 'G' at the position 431 of U67357, corresponding to position 561 (no. 1 is corresponding to the 'A' in the initiation codon in U64511) in the mRNA sequence (Figure 4). But no such deletion was observed in the slightly affected member, neither was in the unrelated healthy individual.

### DISCUSSION

In the present large family a heterozygous deletion of a 'G' in exon 3 of EXT2 gene likely represents the causative defect. It results in frameshift and premature stop codon that would lead to the

*© 2001 Cancer Research Campaign*

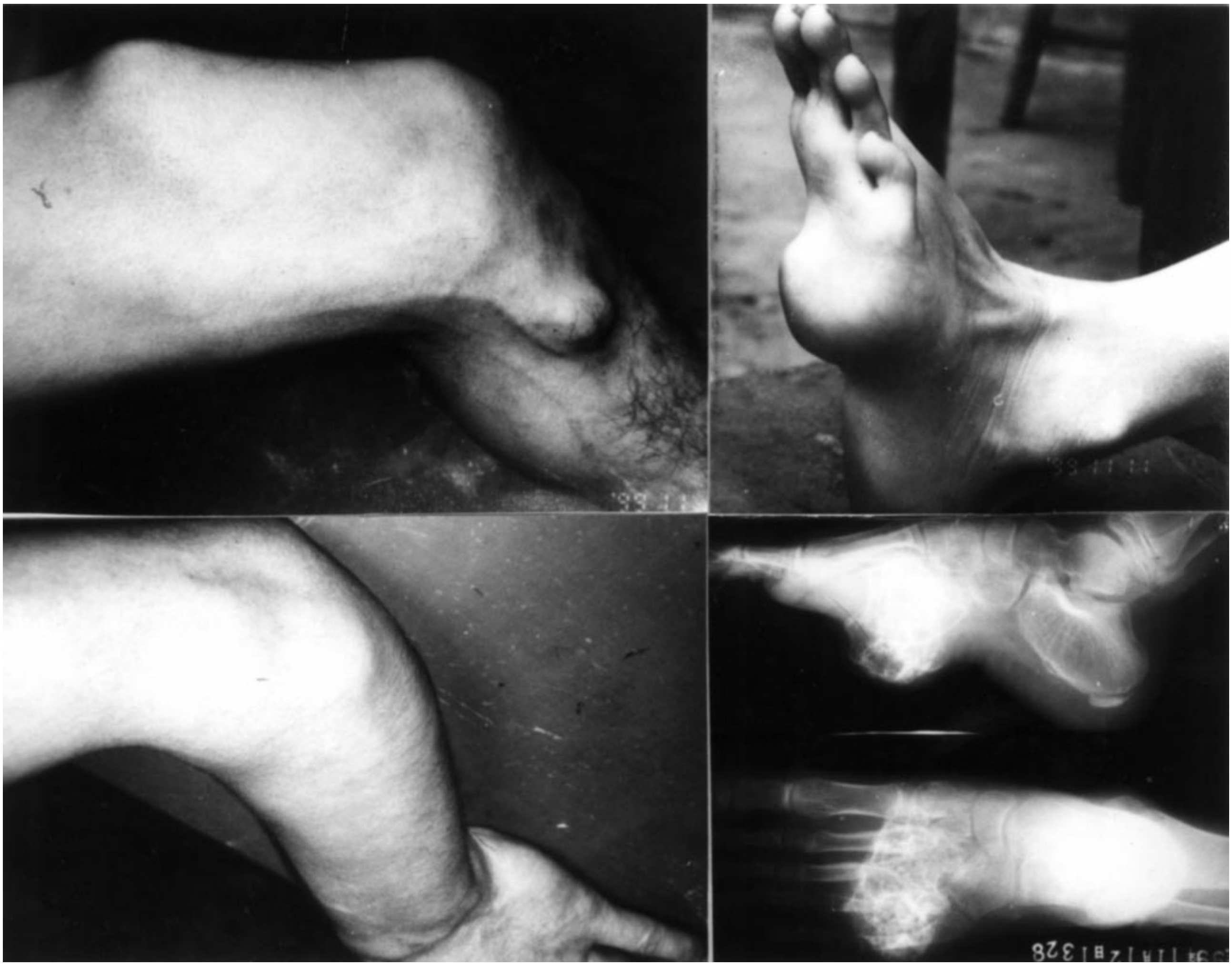

**Figure 2** Local appearance and radiological findings in two affected members. Large and multiple exostosis around the knee and elbow of IV56 (left) and on the foot of VI16 (right)

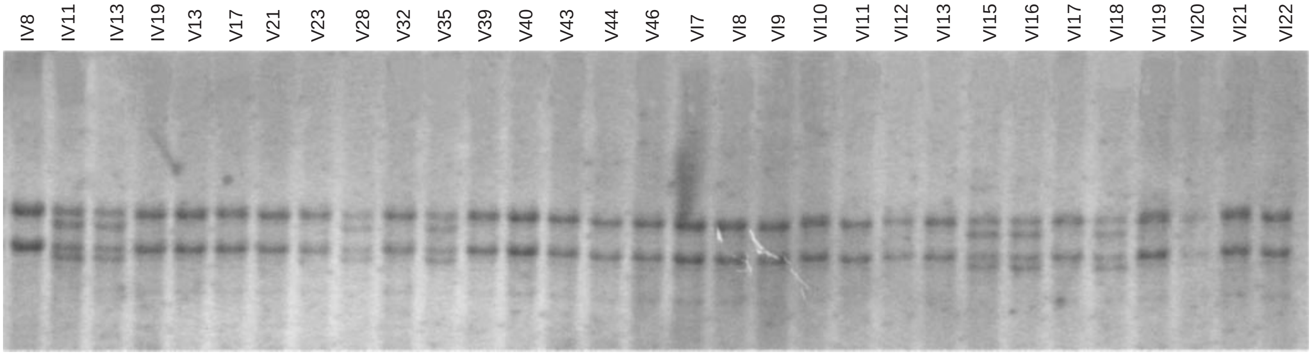

**Figure 3** SSCP of the exon 3 of EXT2 gene in some of the pedigree members. IV13, V28, V35, VI15, VI16 and VI18 are severely affected patients and IV11 is a carrier, and in these persons besides the 2 normal single-stranded bands, 2 additional single-stranded bands are observed. The others are healthy members in the family, in which only 2 single-stranded bands are observed expression of a predicted truncated EXT protein with only 281 amino acids, 437 amino acids shorter than the wild-type protein.

EXT1 and EXT2 genes are putative tumour suppressor genes (Ahn et al, 1995; Hecht et al, 1995; Raskind et al, 1995). It has been reported that proteins encoded by these genes, are endoplasmic reticulum-localized type II transmembrane glycoproteins that possess or are tightly associated with glycosyltransferase activities in the polymerization of heparan sulfate (Lind et al, 1998; McCormick et al, 2000). EXT2 does not harbour significant glycosyltransferase activity in the absence of EXT1. Instead,

© 2001 Cancer Research Campaign 

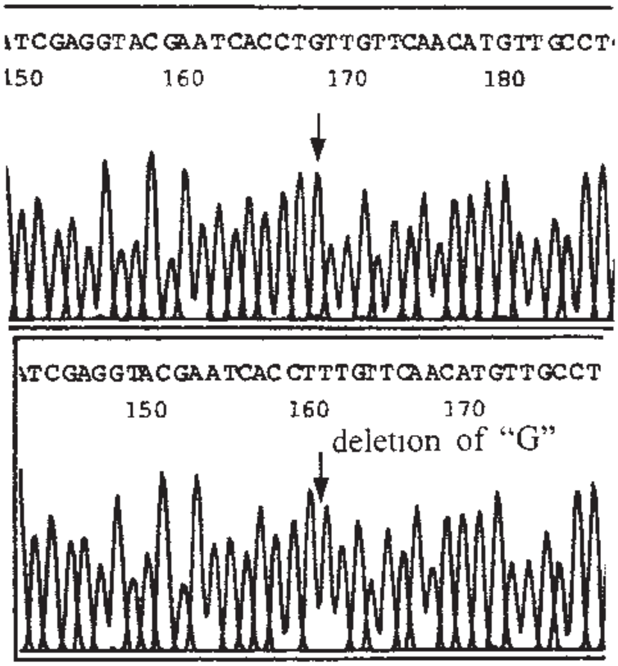

**Figure 4** Sequence analysis of the deletion. This graph illustrates the 'G' deletion in exon 3 of EXT2 gene. The arrow indicates the 'G' in the wild-type allele (upper graph) and is missing in the mutant allele (lower graph) of the severely affected members in the family. PCR products of exon 3 of EXT2 gene in the affected members and normal controls in the family were cloned into pGEM T easy vector, and the double stranded plasmids were sequenced by Sanger method

EXT1 and EXT2 form a hetero-oligomeric complex in vivo that leads to the accumulation of both proteins in the Golgi apparatus. Remarkably, the Golgi-localized EXT1/EXT2 complex possesses substantially high glycosyltransferase activity than EXT1 or EXT2 alone, suggesting that the complex represents the biologically relevant form of the enzyme(s). These can easily explain how inherited mutations in either of the two EXT genes can give rise to hereditary multiple exostoses (McCormick et al, 2000).

Since the identification of EXT1 and EXT2 genes, there have been many different mutations reported. Among them at least 49 different EXT1 and 25 different EXT2 mutations have been detected in EXT patients, and there is evidence that mutations in these 2 genes are responsible for over 70% of the EXT cases (Wuyts and Van Hul, 2000). Among the 49 EXT1 mutations, 9 are nonsense, 21 frameshift and 5 splice site mutations, 2 in-frame deletions with 1 and 5 amino acids respectively, and 12 missense mutations. The 25 EXT2 mutations include 8 nonsense, 11 frameshift, 3 splice site and 3 missense mutations. The deletion of one nucleotide described in present study has not been described before.

It is interesting to note that in the studied family, there are at least 3 affected members who develop only a single exostosis, and 2 of them are in their 30s, the third one is 10. In 2 of these 3 slightly affected patients (V51 and VI25, the third one's blood sample was not available), SSCP analysis of exon 3 failed to detect the same two additional single strand bands as seen in other severely affected ones. This implies that they might be cases of phenocopy, or alternatively, the causative gene abnormality in these 3 individuals may be different from that of the others in the pedigree, although that 2 different mutations in same family is scarce. However, whatever the reason is, the interesting feature makes genetic counselling of the patients more complicated.

According to the genotype-phenotype correlation, the penetrance of EXT in the family is not complete. There is one member (IV11), whose daughter and granddaughter are affected, carries the 2 additional single strand bands in SSCP, hence the mutation, but displays no any visible exostoses.

According to literature, the general recommended treatment to symptomatic exostoses or dislocated radial heads is to remove them. Surgery can improve aesthetic appearance and relieve the pain when done before or after skeletal maturity (Arms et al, 1997; Porter et al, 2000; Vasseur and Fabre, 2000). It seems that the tumour does not interfere with the longevity of the patients and several affected individuals in this family live up to their 80s to 90s. Whether surgical removal of the exostoses is responsible for the progression to malignancy is not clear. It is possible that some unknown factors including some modifying genes may play an important role in the malignant transformation.

### ACKNOWLEDGEMENTS

This work was supported by the grant of NNSFC (No. 39993420) for the research project 'Studies on the Genome Structure and Function in Chinese Nationalities'. We would like to thank our colleagues Yan Peng and Haobo Xie for their participation in this experiment.

### REFERENCES

Ahn J, Lüdecke HJ, Lindow S, Horton WA, Lee B, Wagner MJ, Horsthemke B and Wells DE (1995) Cloning of the putative tumor suppressor gene for hereditary multiple exostoses (EXT1). *Nat Genet* **11**: 137–143

Arms DM, Strecker WB, Manske PR and Schoenecker PL (1997) Management of forearm deformity in multiple hereditary osteochondromatosis. *J Pediatr Orthop* **17**: 450–454

Hecht JT, Hogue D, Strong LC, Hansen MF, Blanton SH and Wagner M (1995) Hereditary multiple exostosis and chondrosarcomas: linkage to chromosome 11 and loss of heterozygosity for EXT-linked markers on chromosomes 11 and 8. *Am J Hum Genet* **56**: 1125–1131

Hennekam R (1991) Hereditary multiple exostoses. *J Med Genet* **28**: 262–266

Lathrop GM, Lalouel JM, Julier C and Ott J (1984) Strategies for multilocus linkage analysis in humans. *Proc Natl Acad Sci USA* **81**: 3443–3446

Le Merrer M, Legeai-Mallet L, Jeannin PM, Horsthemke B, Schinzel A, Plauchu H, Toutain A, Achard F, Munnich A and Maroteaux P (1994) A gene for hereditary multiple exostoses maps to chromosome 19p. *Hum Mol Genet* **3**: 717–722

Lind T, Tufaro F, MaCormick C, Lindahl U and Linholt K (1998) The putative tumor suppressors EXT1 and EXT2 are glycosyltransferases required for the biosynthesis of heparan sulfate. *J Biol Chem* **273**: 26265–26268

McCormick C, Duncan G, Goutsos KT and Tufaro F (2000) The putative tumor suppressors EXT1 and EXT2 form a stable complex that accumulates in the Golgi apparatus and catalyzes the synthesis of heparan sulfate. *Proc Natl Acad Sci USA* **97**: 668–673

Miller SA, Dykes DD and Polesky HF (1988) A simple salting out procedure for extracting DNA from human nucleated cells. *Nucleic Acids Res* **16**: 1215

Porter DE, Emerton ME, Villanueva-Lopez F and Sinpson AH (2000) Clinical and radiographic analysis of osteochondromas and growth disturbance in hereditary multiple exostoses. *J Pedatr Orthop* **20**: 246–250

Raskind WH, Conrad EU, Chansky H and Matsushita M (1995) Loss of heterozygosity in chondrosarcomas for markers 8 and 11. *Am J Hum Genet* **56**: 1132–1139

Schmale G, Conrad E and Raskind W (1994) The natural history of hereditary multiple exostoses. *J Bone Joint Surg Am* **76**: 986–992

Solomon L (1963) Hereditary multiple exostosis. *J Bone Surg* **45B**: 292–304

Stickens D, Clines G, Burbee D, Ramos P, Thomas S, Hogue D, Hecht JT, Lovett M and Evans GA (1996) The EXT2 multiple exostoses gene defines a family of putative tumor suppressor genes. *Nat Genet* **14**: 25–32

Sugiura Y, Sugiura I and Iwata H (1976) Hereditary multiple exostoses: diaphyseal aclasis. *Jpn J Hum Genet* **21**: 149–167

© 2001 Cancer Research Campaign

Tang Y, Xia JH, Zhou JN, Li HJ, Wang DP, Dai HP, Long ZG, Tang BS, Huang L and Deng HX (1998) Localization of the gene for 4 hereditary multiple exostoses families. *I Chuan Hsueh Pao* **25**: 1–7

Van Hul W, Wuyts W, Hendrickx J, Speleman F, Wauters J, De Boulle K, Van Roy N, Bossuyt P and Willems PJ (1998) Identification of a third EXT-like gene (EXTL3) belonging to the EXT gene family. *Genomics* **47**: 230–237

Vasseur MA and Fabre O (2000) Vascular complications of osteochondromas. *J Vasc Surg* **31**: 532–538

Walsh PS, Metzger DA and Higuchi R (1991) Chelex 100 as a medium for simple extraction of DNA for PCR-based typing from forensic material. *Biotechniques* **10**: 506–513

Wicklund CL, Pauli RM, Johnston D and Hecht JT (1995) Natural history study of hereditary multiple exostoses. *Am J Med Genet* **55**: 43–46

Wise CA, Clines GA, Massa H, Trask BJ and Lovett M (1997) Identification and localization of the gene for EXTL, a third member of the multiple exostoses gene family. *Genome Res* **7**: 10–16

Wuyts W, Van Hul W, Wauters J, Nemtsova M, Reyniers E, Van Hul E, De Boulle K, de Vries BB, Hendrickx J, Herrygers I, Bossuyt P, Balemans W, Fransen E, Vits L, Coucke P, Nowak NJ, Shows TB, Mallet L, van den Ouweland AM, McGaughran J, Halley DJ and Willems PJ (1996) Positional cloning of a gene involved in hereditary multiple exostoses. *Hum Mol Genet* **5**: 1547–1557

Wuyts W, Van Hul W, Hendrickx J, Speleman F, Wauters J, De Boulle K, Van Roy N, Van Agtmael T, Bossuyt P and Willems PJ (1997) Identification and characterization of a novel member of the EXT gene family, EXTL2. *Eur J Hum Genet* **5**: 382–389

Wuyts W, Van Hul W, De Boulle K, Hendrickx J, Bakker E, Vanhoenacker F, Mollica F, Ludecke HJ, Sayli BS, Pazzaglia UE, Mortier G, Hamel B, Conrad EU, Matsushita M, Raskind WH and Willems PJ (1998) Mutations in the EXT1 and EXT2 genes in hereditary multiple exostoses. *Am J Hum Genet* **62**: 346–354

Wuyts W and Van Hul W (2000) Molecular basis of multiple exostoses: mutations in the EXT1 and EXT2 genes. *Hum Mutat* **15**: 220–227

*© 2001 Cancer Research Campaign*
